# Supplementary material for: Exceptionally selective voltage-sensor trapping of NaV1.5 channels by Mg-protoporphyrin impairs cancer cell migration
Source: Sci Rep. 2026 Jan 29;16:4085. doi: 10.1038/s41598-026-37492-0 (PMC12855270; doi:10.1038/s41598-026-37492-0)
Supplement: Supplementary file 1 — Supplementary Material 1 [file 41598_2026_37492_MOESM1_ESM.pdf]

Exceptionally selective voltage-sensor trapping of $Na_V1.5$ channels by Mg-protoporphyrin impairs cancer cell migration

Supplementary Material

Mahdi Jamili[a], Marwa Ahmed[a], Alisa Bernert[a], Johann Rößler[a], Guido Gessner[a], Roland Schönherr[a], Toshinori Hoshi[b] & Stefan H. Heinemann[a,*]

[a] *Center for Molecular Biomedicine, Department of Biophysics, Friedrich Schiller University Jena and Jena University Hospital, Jena, Germany*

[b] *Department of Physiology, University of Pennsylvania, Philadelphia, PA 19104-6085, USA*

* Corresponding author
Center for Molecular Biomedicine, Department of Biophysics, Friedrich Schiller University Jena and Jena University Hospital, Hans-Knöll-Straße 2, 07745 Jena, Germany
stefan.h.heinemann@uni-jena.de
ORCID: 0000-0002-4144-0251

Supplementary Tables

Supplementary Table 1 | Parameters describing the voltage dependence of activation and inactivation, as well as the remaining current at -30 mV (*Rem. I*) after the application of 100 nM MgPpIX for Na_V-channel isoforms and mutants.

Channel type	V_m (mV)	k_m (mV)	*n*	V_h (mV)	k_h (mV)	*n*	Rem. *I* in 100 nM MgPpIX (%)	*n*
$hNa_V1.5$	-55.6 ± 0.6	8.8 ± 0.2	39	-94.2 ± 0.8	5.7 ± 0.1	40	3.4 ± 0.4	10
$nhNa_V1.5$	-48.9 ± 2.5	9.8 ± 0.7	6	-90.3 ± 1.8	5.7 ± 0.2	6	3.8 ± 0.7	5
$mNa_V1.5$	-59.5 ± 0.9	8.7 ± 0.2	19	-94.7 ± 0.9	5.6 ± 0.1	21	35.1 ± 2.8	9
$hNa_V1.2$	-40.1 ± 0.9	8.0 ± 0.5	9	-70.1 ± 1.5	6.4 ± 0.6	9	105.7 ± 2.0	5
$hNa_V1.4$	-40.8 ± 0.6	8.3 ± 0.4	9	-76.5 ± 0.6	5.6 ± 0.2	10	101.8 ± 3.3	5
$hNa_V1.7$	-40.5 ± 1.2	8.6 ± 0.3	11	-85.5 ± 1.1	5.8 ± 0.2	11	106.5 ± 3.7	6
$hNa_V1.5$ mutants:								
C373Y	-54.1 ± 1.4	8.0 ± 0.4	5	-92.9 ± 1.4	5.5 ± 0.2	5	5.4 ± 1.2	5
E737A	-45.1 ± 1.9	9.4 ± 0.3	5	-94.6 ± 1.8	5.4 ± 0.2	5	13.3 ± 2.5	5
N740P	-57.0 ± 1.5	8.8 ± 0.5	10	-96.2 ± 1.8	6.0 ± 0.2	11	5.6 ± 0.6	5
N740Q	-53.3 ± 1.2	8.6 ± 0.4	5	-91.1 ± 0.9	5.7 ± 0.3	5	6.5 ± 0.7	5
E743A	-57.7 ± 0.9	8.4 ± 0.5	6	-98.2 ± 1.6	5.5 ± 0.2	7	5.1 ± 1.1	7
S743E	-55.4 ± 1.6	8.5 ± 0.3	5	-95.1 ± 1.7	6.0 ± 0.4	5	32.4 ± 3.3	5
E746S	-49.3 ± 2.2	10.0 ± 0.8	5	-91.0 ± 1.7	5.3 ± 3.1	5	4.1 ± 1.2	5
E746K	-59.8 ± 1.5	8.6 ± 0.5	6	-100.3 ± 1.4	5.4 ± 0.2	7	7.4 ± 0.9	7
E795A	-48.0 ± 1.2	12.9 ± 0.3	8	-96.9 ± 1.8	5.7 ± 0.2	8	100.0 ± 2.3	5
R800A	-55.6 ± 2.4	10.3 ± 0.8	5	-100.4 ± 3.2	5.6 ± 3.2	5	6.8 ± 1.8	5
R800D	-63.2 ± 2.2	8.9 ± 0.4	5	-103.0 ± 1.7	5.5 ± 0.4	5	15.2 ± 3.3	6
S802G	-54.2 ± 2.1	9.4 ± 0.8	5	-97.1 ± 1.7	6.0 ± 0.4	5	27.7 ± 4.1	5
S802E	-64.7 ± 1.3	9.3 ± 9.1	5	-108.5 ± 3.0	6.4 ± 0.4	5	26.7 ± 2.6	5
N803G	-51.9 ± 0.9	9.6 ± 0.5	5	-95.4 ± 0.9	5.5 ± 0.1	5	93.8 ± 1.2	5
N803A	-51.9 ± 1.8	9.6 ± 0.2	5	-98.3 ± 1.3	6.1 ± 0.5	5	90.3 ± 4.6	6
N803Q	-56.2 ± 1.1	8.1 ± 0.3	5	-94.4 ± 2.0	5.5 ± 0.3	5	5.5 ± 2.1	5
N803S	-55.9 ± 1.0	9.5 ± 0.5	8	-98.0 ± 1.7	5.9 ± 0.2	8	89.0 ± 2.3	6
N803F	-42.3 ± 1.2	13.8 ± 0.2	8	-98.9 ± 1.3	5.6 ± 0.2	8	80.8 ± 2.9	7
N803H	-54.1 ± 1.7	10.4 ± 0.3	5	-98.3 ± 2.9	6.4 ± 0.5	5	6.5 ± 1.0	8
N803D	-60.9 ± 0.6	7.7 ± 0.6	7	-98.4 ± 1.9	5.6 ± 0.3	7	94.5 ± 2.0	5
N803R	-54.2 ± 2.0	9.8 ± 0.6	5	-101.2 ± 2.6	6.2 ± 0.2	5	12.2 ± 1.5	5
N803K	-50.6 ± 0.9	9.5 ± 0.2	5	-94.6 ± 3.0	6.8 ± 0.5	5	18.5 ± 1.9	5
S802E:N803G	-60.1 ± 1.7	7.9 ± 0.6	5	-100.3 ± 1.3	6.0 ± 0.4	5	90.1 ± 2.4	5
R808A	-44.8 ± 1.1	11.8 ± 0.3	5	-100.0 ± 0.8	6.8 ± 0.5	5	58.5 ± 5.5	5
$hNa_V1.7$ mutants:								
E829S	-38.2 ± 0.3	10.6 ± 0.2	5	-90.7 ± 1.7	6.1 ± 0.3	5	97.3 ± 10.4	5
G830N	-40.3 ± 2.2	9.9 ± 0.6	6	-90.1 ± 1.7	5.9 ± 0.4	6	106.1 ± 5.1	6
E829S:G830N	-58.5 ± 1.1	9.2 ± 0.4	10	-98.5 ± 0.7	5.8 ± 0.3	10	7.5 ± 1.4	5

Supplementary Figures

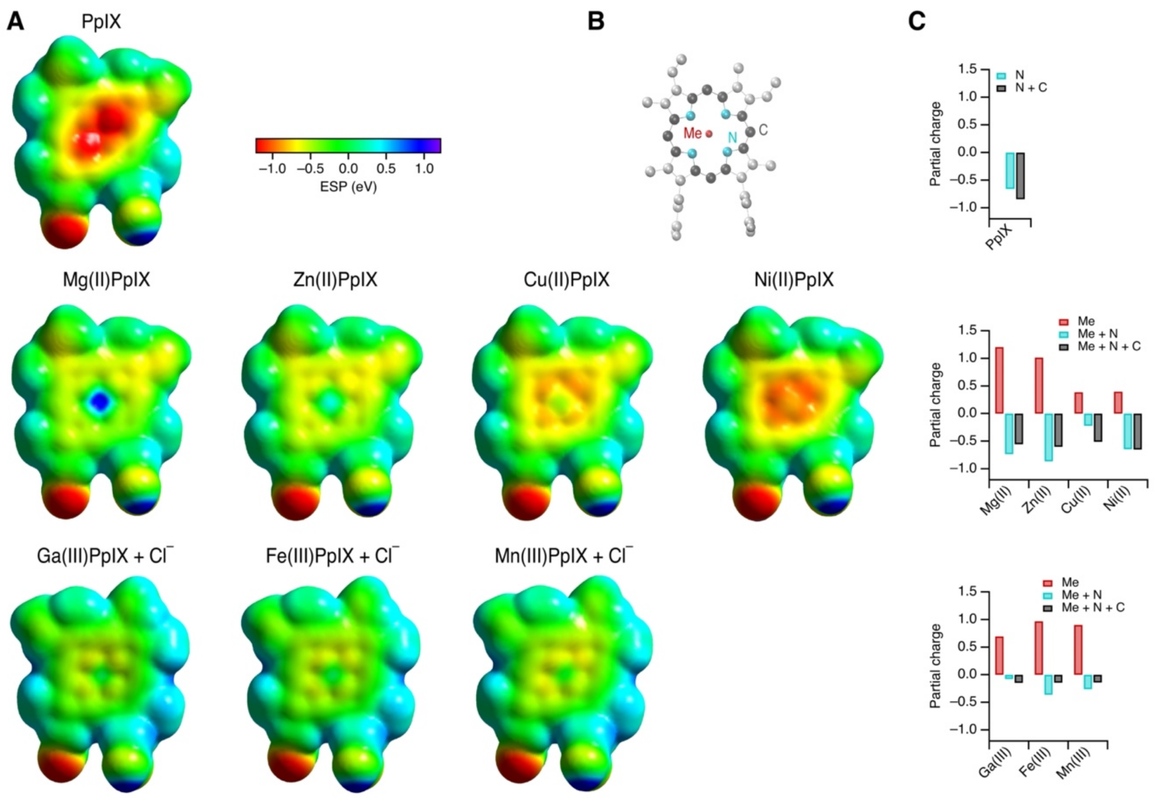

Supplementary Fig. 1 | Electrostatic surface potential (ESP) of MePpIX. A Results of the DFT (density functional theory) calculations with Gaussian 16, using the aug-cc-pVDZ basis set (augmented double Dunning's correlation consistent basis set) with water as the solvent. ESPs mapped onto electron density isosurfaces of 0.0004 atomic units are shown in electron volts (eV) according to the color map. The structures are grouped according to no metal center (PpIX, *top*), divalent ion configuration (*middle*), and trivalent ion configuration (*bottom*). In the latter cases, the structures also contain a chloride ion on the reverse side to compensate for the extra charge and to make the surface potentials comparable. In all cases, each of the carboxylate groups of the PpIX rings was saturated with an extra H^+. The optimization was performed for Cu(II)PpIX and Zn(II)PpIX with default, and for the remaining variants with a loose convergence criterion. **B** MgPpIX structure as an example to indicate which atoms were used to calculate the summed partial charges, as shown in **C**. **C** Partial charges of the metal center (red), the metal center plus the surrounding ring of nitrogen atoms (cyan), and also including the carbon atoms of the PpIX ring (dark gray). ESP charges were determined from the Gaussian checkpoint files using MK (Merz-Singh-Kollman) population analysis and UFF (Universal Force Field) radii (Iop 6/41=10, 6/42=10).

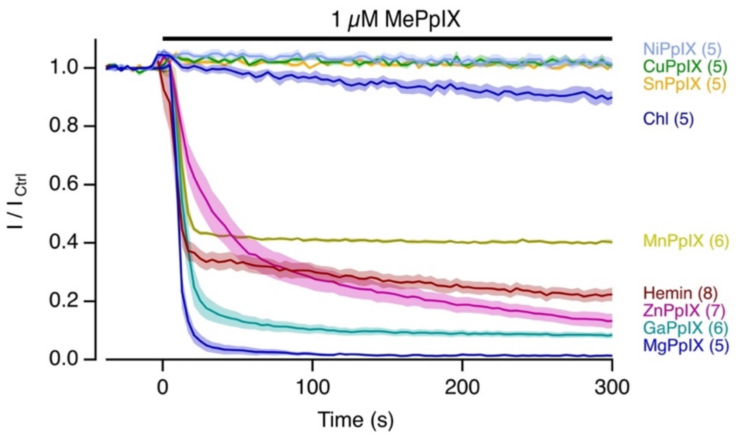

Supplementary Fig. 2 | Kinetics of hNa$_V$1.5 inhibition by MePpIX. The time course of the normalized peak current at -30 mV with the application of the indicated metal protoporphyrins (MePpIXs) or chlorophyll-A (Chl) at 1 µM. The thick lines denote mean values, and sem is indicated by shading, *n* in parentheses.

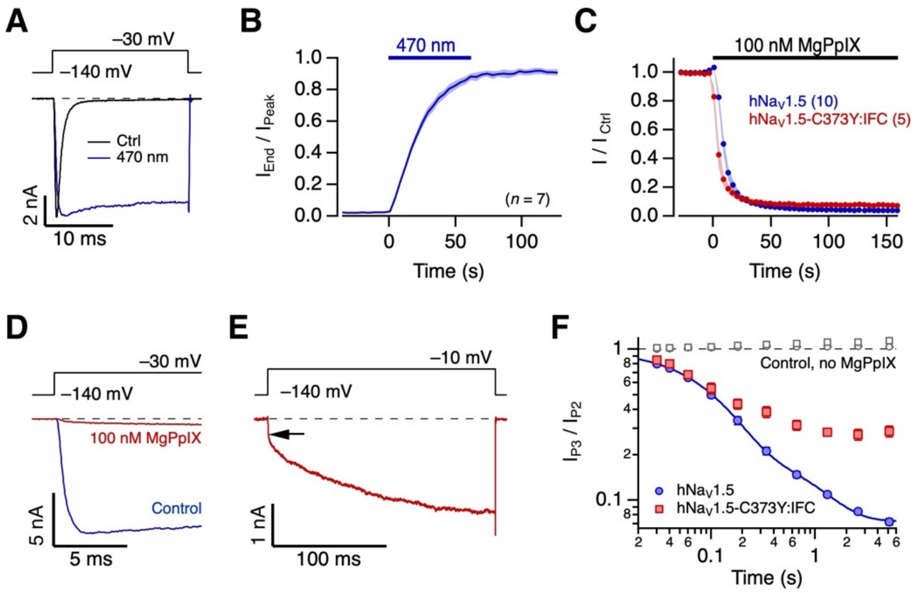

Supplementary Fig. 3 | Light-inducible non-inactivating hNa$_V$1.5 channels. Experiments were carried out with hNa$_V$1.5-C373Y:IFC, i.e., a mutant lacking a cysteine residue in the pore but possessing a cysteine instead of methionine in the inactivation domain "IFM" between domains III and IV, referred to as "IFC" to reflect the mutation M1487C. The mutant was expressed in HEK293T cells, and currents were measured in the whole-cell mode. The pipette solution was supplemented with 250 µM lucifer yellow. **A** Representative current recording for the indicated step depolarization under control conditions (black) and after blue-light illumination of the cell filled with lucifer yellow (blue). **B** Mean time course of the current at the end of a 20-ms depolarization divided by the peak current (to serve as an index of inactivation loss) as a function of time. The horizontal bar indicates the illumination of the cell through a 20x objective with light from a 470-nm LED. The thick line represents the mean, and sem is indicated by shading. The loss of inactivation proceeded with a time constant of about 15 s. **C** Time course of normalized peak current at -30 mV with the application of 100 nM PpIX for hNa$_V$1.5 (inactivating, blue) and hNa$_V$1.5-C373Y:IFC channels after light-induced inactivation removal (red). Straight lines connect the data points for clarity. **D** Superposition of non-inactivating hNa$_V$1.5-C373Y:IFC currents before (blue) and after application of 100 nM MgPpIX (red). **E** Current trace under the conditions shown in **D** for 200 ms visualizing the reversal of channel inhibition at -10 mV. The arrow indicates the approximate instantaneous current level before reverse use-dependence occurs. **F** Kinetics of channel block after partial relief from inhibition (as in Fig. 2E, with double-exponential fit for hNa$_V$1.5). Open symbols refer to control measurements in the absence of MgPpIX.

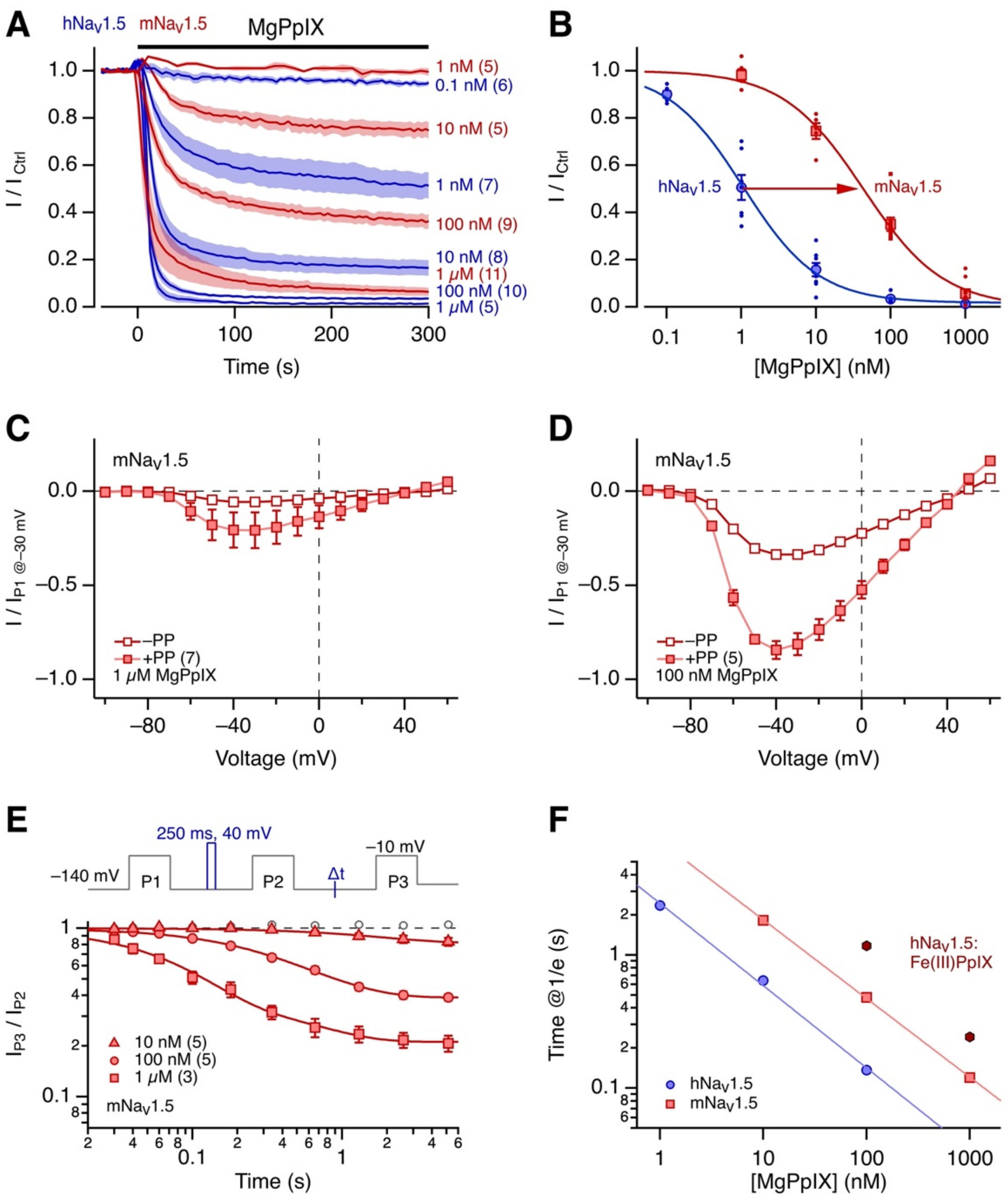

Supplementary Fig. 4 | Inhibition of mouse Na$_V$1.5 channels by MgPpIX. A Time course of peak current (at -30 mV) inhibition by the indicated concentrations of MgPpIX for human hNa$_V$1.5 (blue, from Fig. 1) and mouse mNa$_V$1.5 (red). Thick lines represent means and sem is indicated by shading, *n* in parentheses. **B** Concentration-response of the normalized current after 300 s of MgPpIX application with superimposed Hill fits (Eq. 4). Data for hNa$_V$1.5 are identical to those of Fig. 1; the results for mNa$_V$1.5 are: IC_{50} = 42.8 ± 5.0 nM, n_H = 0.81 ± 0.07, and a_∞ was constrained to 0. **C** Current–voltage relationships in the presence of 1 µM MgPpIX, normalized to the value obtained at -30 mV prior to MgPpIX application. Open symbols represent values of the first depolarizing pulse (P1), while filled symbols originate from the recordings following a prepulse to -10 mV for 100 ms (P2). Straight lines connect data points for clarity. **D** As in **C** but for 100 nM MgPpIX. **E** Kinetics of the onset of current inhibition after prepulse-induced reversal for mNa$_V$1.5 at the indicated MgPpIX concentrations. Curves are the results of double-exponential fits. Open symbols refer to control conditions in the absence of MgPpIX. **F** From experiments as shown in **E** for mNa$_V$1.5 and Fig. 2E for hNa$_V$1.5, the time needed to inhibit the channels to 1/e (37%) is plotted as a function of MgPpIX concentration. Results for hNa$_V$1.5 and hemin (Fe(III)PpIX) are also indicated. In this log-log

presentation, the re-inhibition kinetics are linear, with the characteristic time becoming about 4.3-times smaller at 10-fold concentration increase. For comparison, the reestablishment of the inhibition was about 8 times slower for Fe(III)PpIX. A comparison of human and mouse $Na_V1.5$ revealed a roughly 3.3-fold faster re- inhibition by MgPpIX for the human isoform.

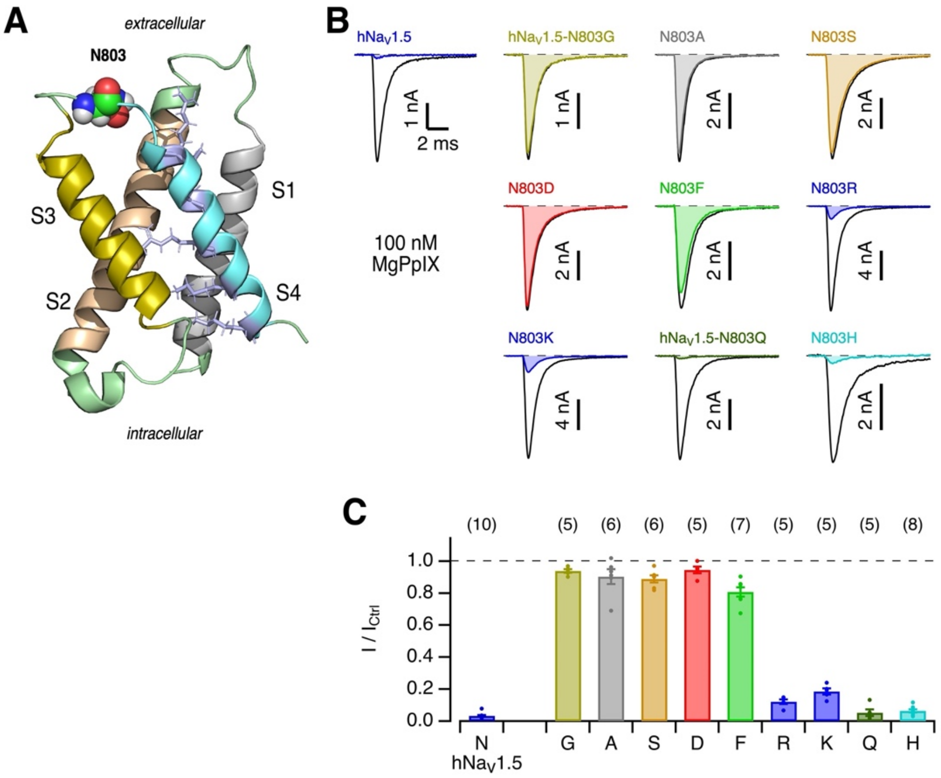

Supplementary Fig. 5 | Mutagenesis of hNa$_V$1.5 at site N803. A Structure of the hNa$_V$1.5 domain-II voltage sensor (from PDB: 6LQA) with residue N803 highlighted: carbon, green; nitrogen, blue; oxygen, red; hydrogen, grey. Positively charged residues of S4 are shown as sticks (light blue). **B** Representative current recordings at -30 mV for the indicated channel types and mutants before (black) and 5 min after the application of 100 nM MgPpIX (colored). **C** Mean fractional peak current at -30 mV remaining 5 min after the application of 100 nM MgPpIX for wild type hNa$_V$1.5 and variants mutated at site N803. Data are means ± sem (*n* in parentheses, individual results indicated as dots).

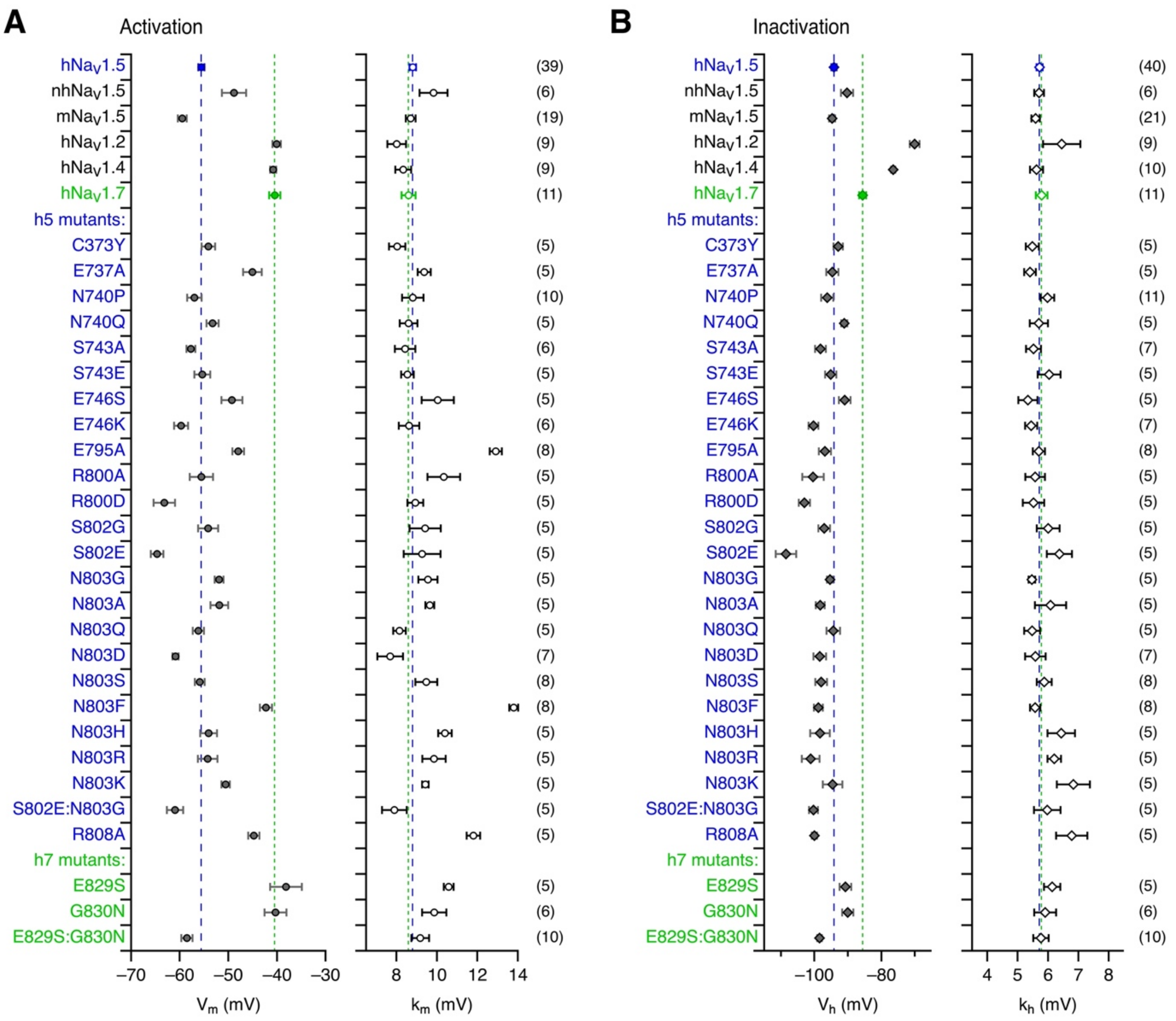

Supplementary Fig. 6 | Parameters describing the voltage dependence of activation and inactivation of Na_V channels and their mutants. A V_m and k_m as results of the analysis of current–voltage relationships (Eq. 1) for the indicated Na_V channel types and mutants. **B** V_h and k_h values according to Eq. 2 describing the voltage dependence of channel inactivation caused by 500-ms prepulses. Data are means ± sem with *n* in parentheses. Most notably, mutation S802E caused a marked left-shift in the voltage dependence of activation and inactivation in $hNa_V1.5$. Moreover, only the combination of the mutations E829S and G830N in $hNa_V1.7$, but not the individual mutations, altered the half-maximal activation voltage (V_m) from -40.5 ± 0.1 mV (n = 11) for $hNa_V1.7$ to -58.5 ± 0.1 mV (n = 10), which is close to the value obtained for $hNa_V1.5$ (-55.6 ± 0.1 mV, n = 39).

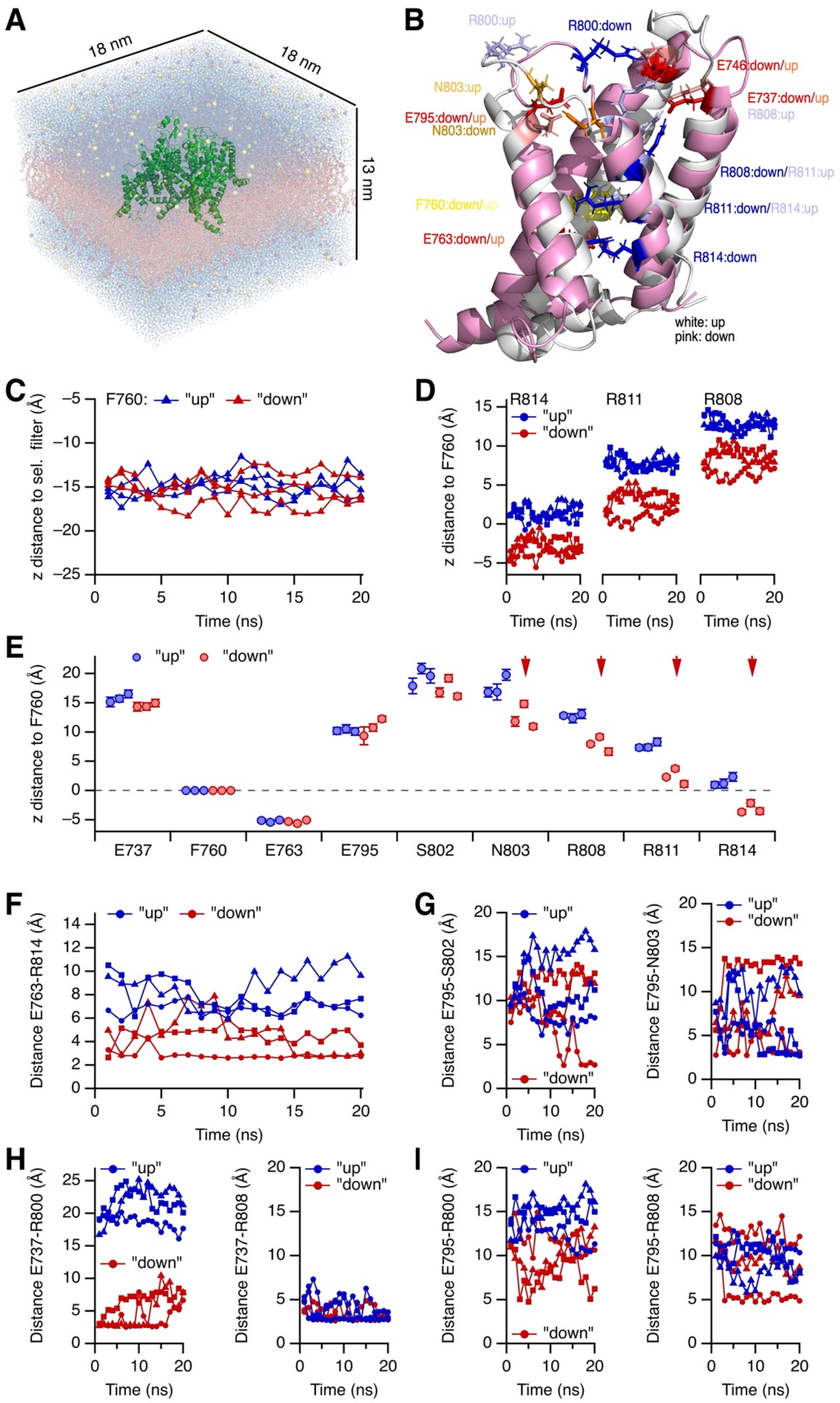

Supplementary Fig. 7 | Structural model of hNa$_V$1.5 with VSD II in a deactivated position.
A Molecular dynamics simulation box containing the channel protein (green), a lipid bilayer (wheat), water molecules (blue), and ions (K^+, purple; Cl^- yellow) with a total of about 470,000 atoms. Periodic boundary conditions were applied during simulation. **B** Superposition of VSD II in “up” (white) and

“down” (pink) configuration. The “up” configuration is the structure as obtained from the cryo-EM data (PDB: 6LQA), the deactivated “down” configuration was obtained by aligning VSD II to the voltage sensor of Kv7.1 channels with subsequent energy minimization. For the superposition, structures were aligned according to the S6 segments (CA atoms only) of all four domains. **C**-**I** Distance measurements of various residues from each three MD simulations of 20 ns in the “up” and “down” configuration of VSD II. **C** z distance of the β carbon (CB) of F760 relative to the mean CB position of the selectivity filter “DEKA” (D372, E901, K1419, A1711) from all four domains, indicating the relative stability of the F760 position within the channel protein. **D** z distance of the CB carbons of R814, R811, and R808 relative to CB of F760. **E** Average values (means of the structures from 10-20 ns, ±s.d.) of the z distances of the indicated residues (always the CB carbon) to F760/CB for three simulation systems in the “up” and three in the “down” configuration. **F** Minimum distance of the amide groups of R814 (NH1, NH2) to the oxygens of E763 (OE1, OE2); R814 and E763 form an ion–ion bond in the “down” configuration. **G** Distances of E795 to S802 (*left*) and N803 (*right*). **H** Distances of E737 to R800 (*left*) and R808 (*right*). **I** Distances of E795 to R800 (*left*) and R808 (*right*).

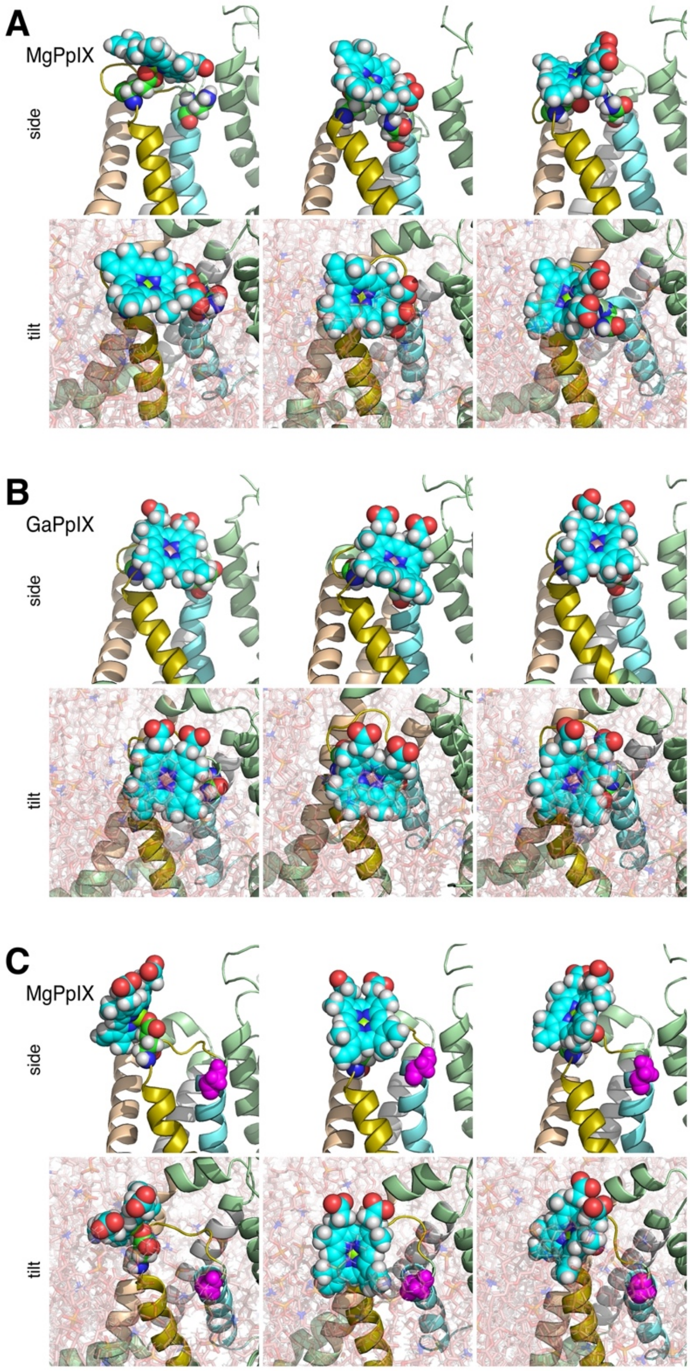

Supplementary Fig. 8 | Possible binding configuration of MePpIX at the voltage sensor of domain II. A-C Three snapshots, one from each of the three molecular dynamics simulation runs, as detailed in Fig. 7 with MgPpIX (**A**, **C**) or GaPpIX (**B**) placed near VSD II. Channel structures are hNa$_V$1.5 with VSD II in a deactivated "down" position (**A**, **B**) and the same for channel mutant N803G (**C**). *top*, Presentations of VSD II in side view without water, ions, and lipids. *bottom*, The same structures as in the *top* panels but including lipids, and tilted by about 45°. Residues E795 and N803 are shown as spheres with carbon atoms in green. G803 (in **C**) is shown in magenta. The carbon atoms of the MePpIX are shown in cyan.

Supplementary Movies

1) Three MD simulations of $hNa_V1.5$ with VSD II in a “down” position and MgPpIX.
   E795 is shown as spheres with atom colors, N803 in orange.
2) Three MD simulations of $hNa_V1.5$ with VSD II in a “down” position and GaPpIX.
   E795 is shown as spheres with atom colors, N803 in orange.
3) Three MD simulations of $hNa_V1.5$ with VSD II in a “down” position and NiPpIX.
   E795 is shown as spheres with atom colors, N803 in orange.
4) Three MD simulations of $hNa_V1.5$-N803G with VSD II in a “down” position and MgPpIX.
   E795 is shown as spheres with atom colors, G803 in orange.
5) Three MD simulations of $hNa_V1.5$-E795A with VSD II in a “down” position and MgPpIX.
   A795 is shown as spheres with atom colors, N803 in orange.

(All files are integrated in one pptx document.)
