# Supplementary material for: Exceptionally selective voltage-sensor trapping of NaV1.5 channels by Mg-protoporphyrin impairs cancer cell migration
Source: Sci Rep. 2026 Jan 29;16:4085. doi: 10.1038/s41598-026-37492-0 (PMC12855270; doi:10.1038/s41598-026-37492-0)
Supplement: Supplementary file 2 — Supplementary Material 2 [file 41598_2026_37492_MOESM2_ESM.zip › MgPpIX_Movies.pptx]

## Slide 1
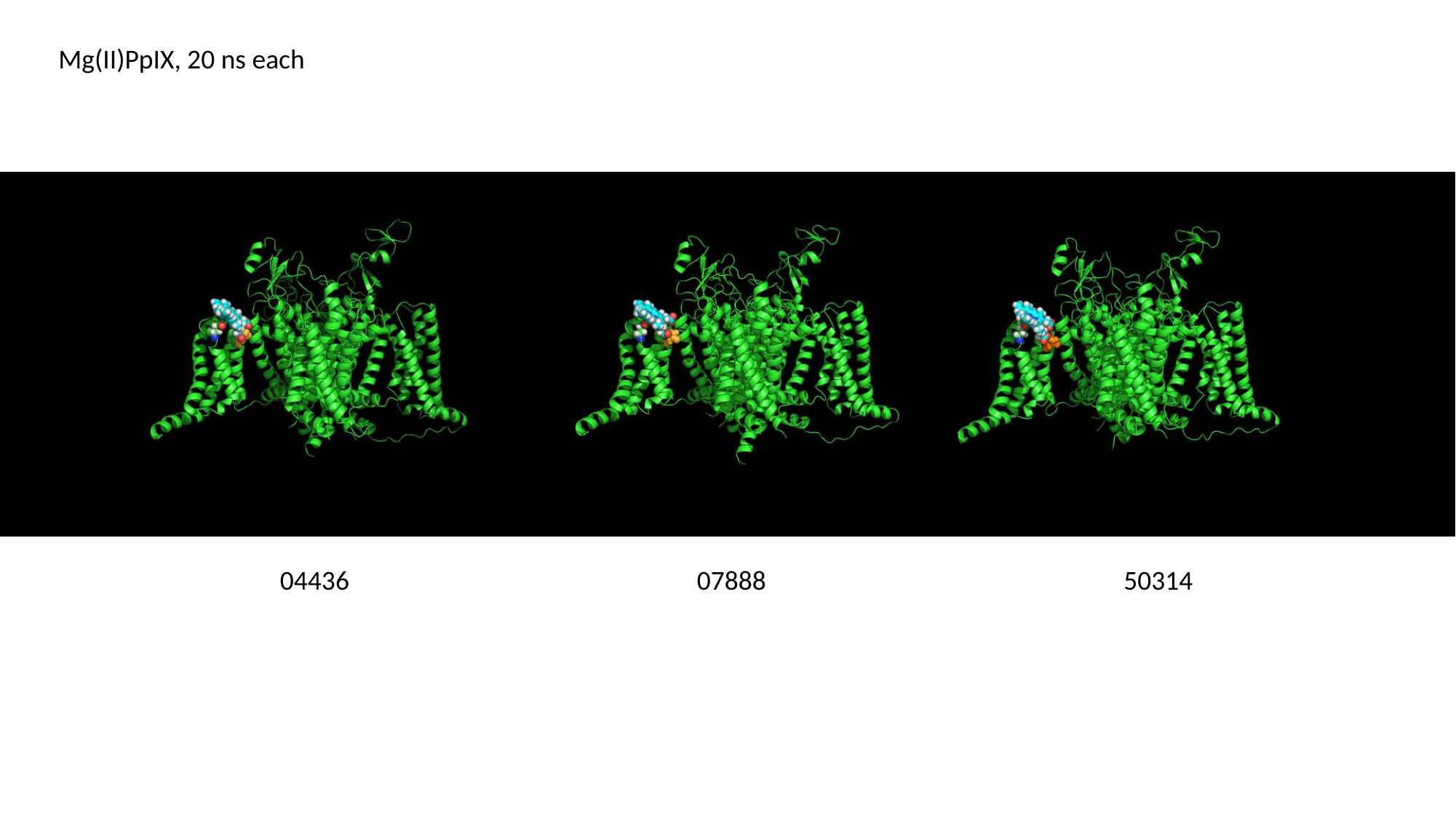

Mg(II)PpIX, 20 ns each
04436
07888
50314

## Slide 2
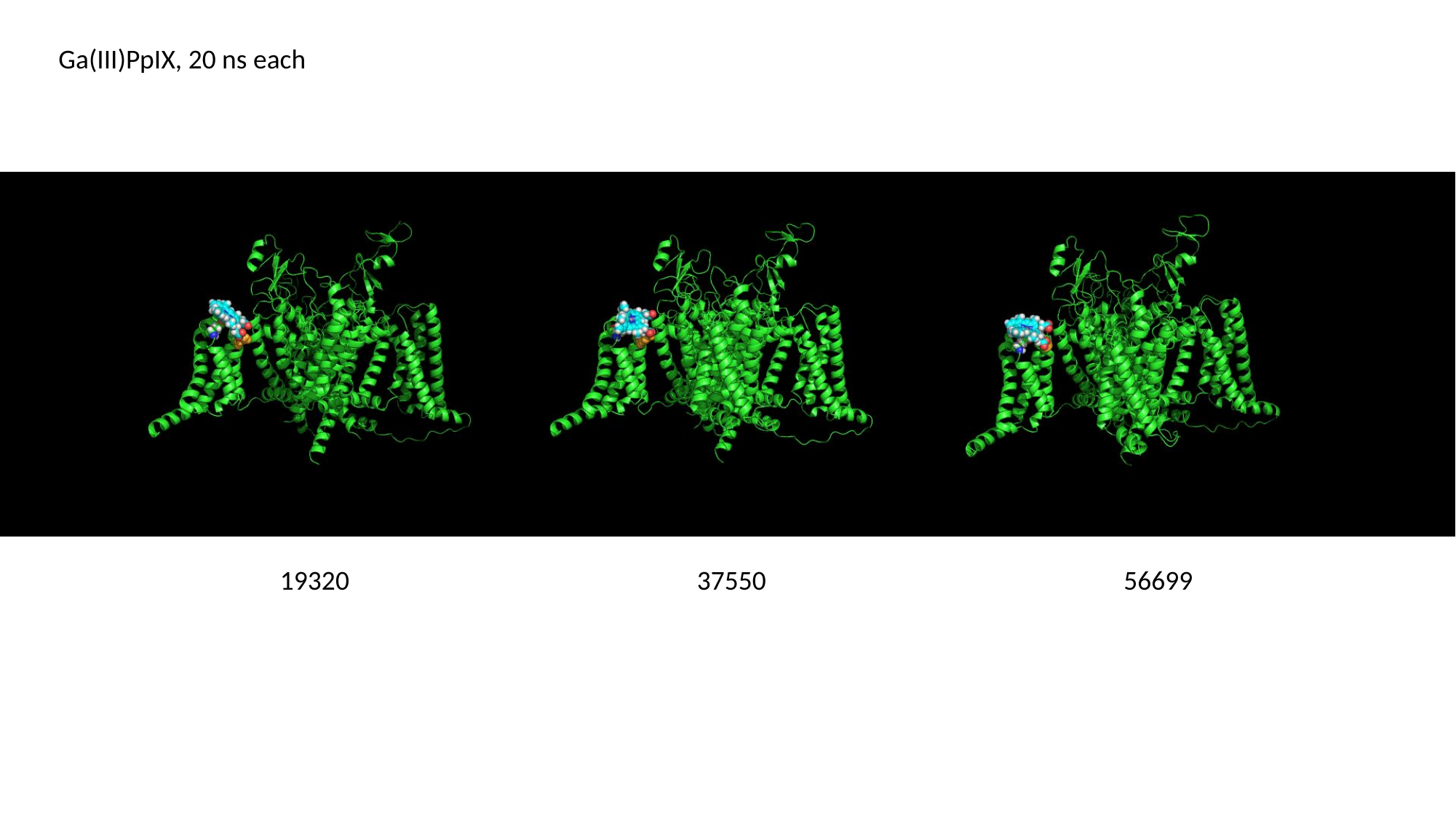

Ga(III)PpIX, 20 ns each
19320
37550
56699

## Slide 3
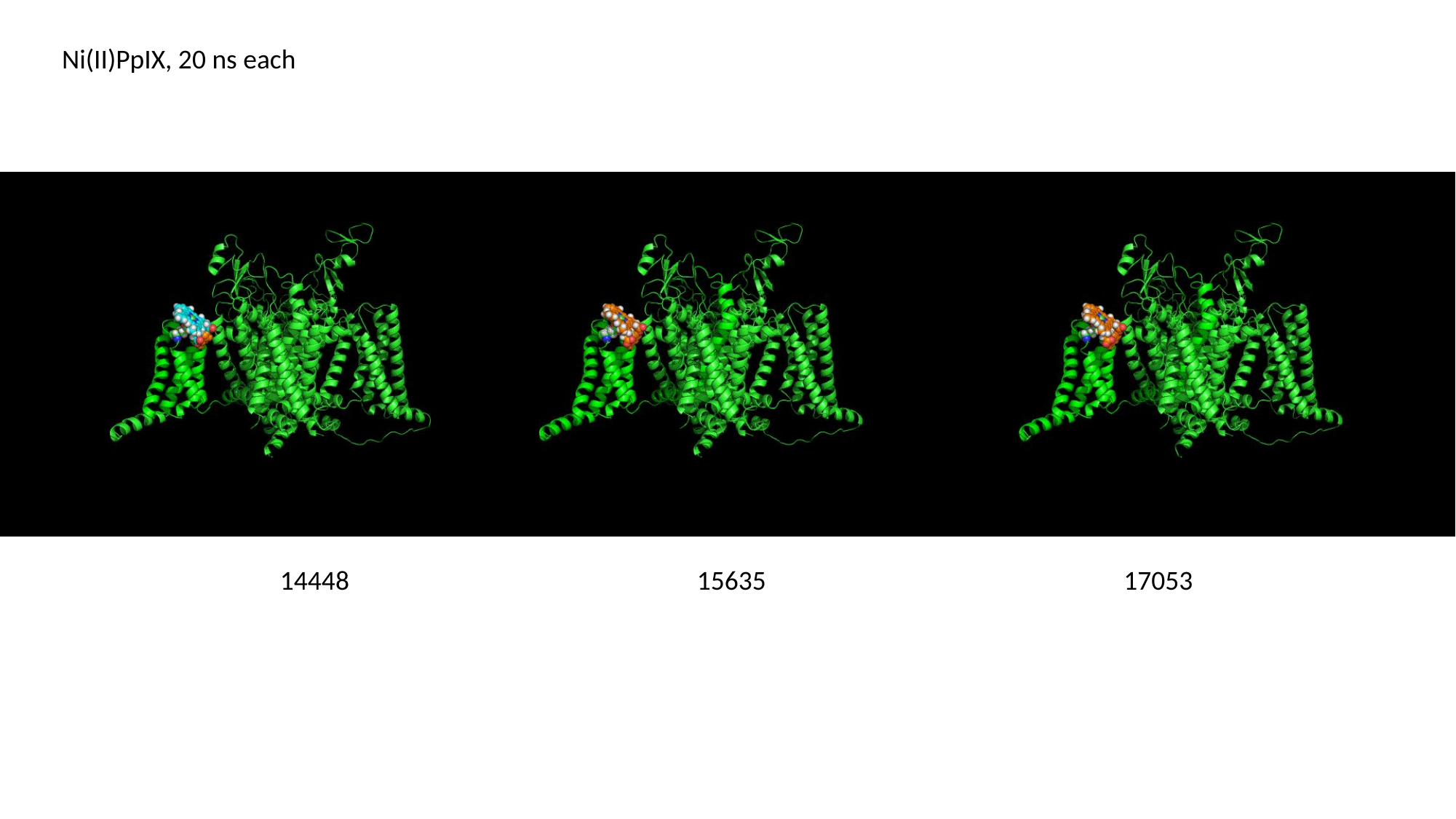

Ni(II)PpIX, 20 ns each
14448
15635
17053

## Slide 4
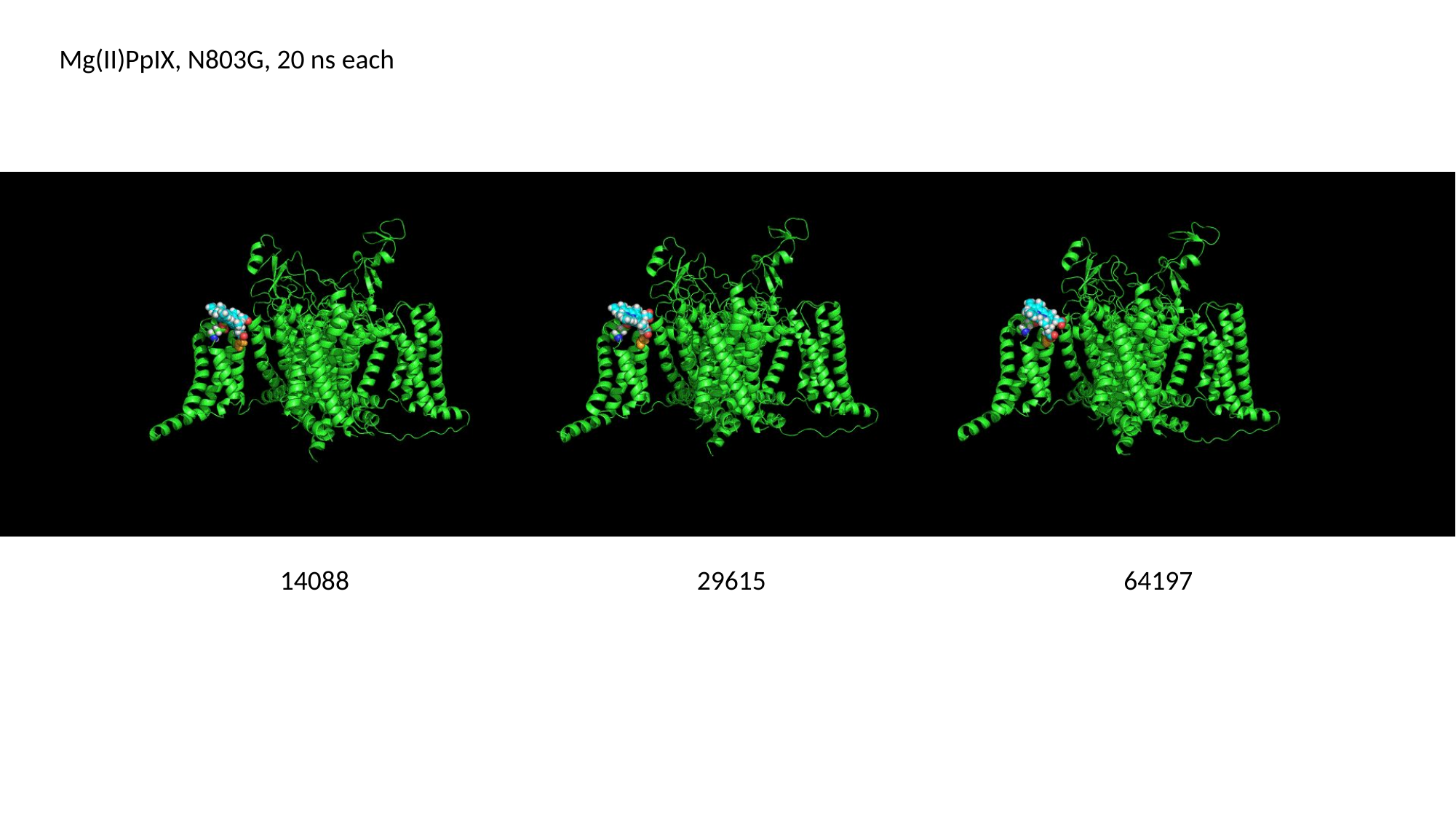

Mg(II)PpIX, N803G, 20 ns each
14088
29615
64197

## Slide 5
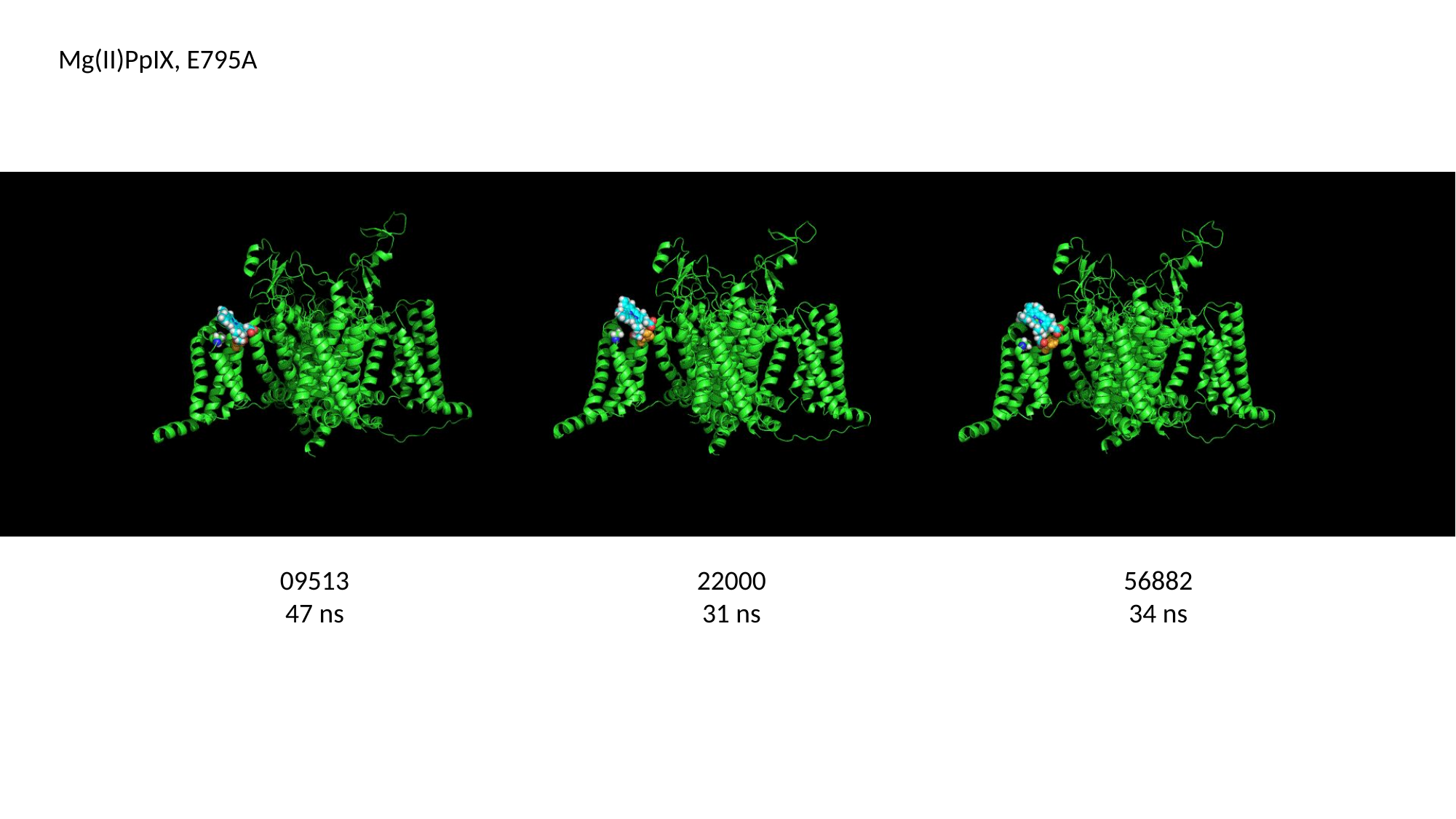

Mg(II)PpIX, E795A
09513
47 ns
22000
31 ns
56882
34 ns
